# Red LED photobiomodulation reduces pain hypersensitivity and improves sensorimotor function following mild T10 hemicontusion spinal cord injury

**DOI:** 10.1186/s12974-016-0679-3

**Published:** 2016-08-26

**Authors:** Di Hu, Shuyu Zhu, Jason Robert Potas

**Affiliations:** 1 The John Curtin School of Medical Research, The Australian National University, Building 131, Garran Rd, Canberra, ACT 2601 Australia; 2 ANU Medical School, The Australian National University, Canberra, ACT 2601 Australia

**Keywords:** Photobiomodulation, Light therapy, M2 macrophage polarization, Allodynia, Neuropathic pain, 670 nm

## Abstract

**Background:**

The development of hypersensitivity following spinal cord injury can result in incurable persistent neuropathic pain. Our objective was to examine the effect of red light therapy on the development of hypersensitivity and sensorimotor function, as well as on microglia/macrophage subpopulations following spinal cord injury.

**Methods:**

Wistar rats were treated (or sham treated) daily for 30 min with an LED red (670 nm) light source (35 mW/cm^2^), transcutaneously applied to the dorsal surface, following a mild T10 hemicontusion injury (or sham injury). The development of hypersensitivity was assessed and sensorimotor function established using locomotor recovery and electrophysiology of dorsal column pathways. Immunohistochemistry and TUNEL were performed to examine cellular changes in the spinal cord.

**Results:**

We demonstrate that red light penetrates through the entire rat spinal cord and significantly reduces signs of hypersensitivity following a mild T10 hemicontusion spinal cord injury. This is accompanied with improved dorsal column pathway functional integrity and locomotor recovery. The functional improvements were preceded by a significant reduction of dying (TUNEL^+^) cells and activated microglia/macrophages (ED1^+^) in the spinal cord. The remaining activated microglia/macrophages were predominantly of the anti-inflammatory/wound-healing subpopulation (Arginase1^+^ED1^+^) which were expressed early, and up to sevenfold greater than that found in sham-treated animals.

**Conclusions:**

These findings demonstrate that a simple yet inexpensive treatment regime of red light reduces the development of hypersensitivity along with sensorimotor improvements following spinal cord injury and may therefore offer new hope for a currently treatment-resistant pain condition.

## Background

The experience of pain serves as an essential survival mechanism that motivates us to protect ourselves from harm; however, following spinal cord injury, the development of treatment-resistant neuropathic pain often ensues, bringing no advantage to the sufferer but severely reducing the quality of life. Chronic pain affects a vast sector of the population for which the socioeconomic cost exceeds that of heart disease, cancer and diabetes [1]; thus, successfully treating neuropathic pain would bring significant benefits.

The non-invasive application of light, at wavelengths that penetrate transcutaneously [2], has begun to emerge as a potential therapy for improving functional outcomes from a variety of neural injuries [3]. Photobiomodulation with wavelengths ranging from 630 to 1100 nm has demonstrated positive effects in animal models of neurodegenerative diseases such as Alzheimer’s [4] and Parkinson’s [5], genetic models of dementia [6], as well as acute nervous injuries to the retina [7–9], optic nerve [9, 10], sciatic nerve [11–15] and spinal cord [16]. In humans, photobiomodulation has been reported to be effective against a variety of pain conditions including mucositis [17], carpel tunnel syndrome [18–20], orthodontic pain [21], temporomandibular joint pain [22], neck pain [23] and neuropathic pain resulting from amputation [24].

Inflammatory mediators have long been implicated in the development and maintenance of pain [25–28]. These chemical mediators are controlled by a variety of immune cells including the balance of pro- and anti-inflammatory microglia/macrophage subpopulations [29–35]. As in non-neural tissues, macrophages can be activated by T helper cell type 1 (Th1) or type 2 (Th2) to generate opposing immune responses following spinal cord injury [30, 31]. Th1-activated microglia/macrophages (M1) have been considered potentially damaging to healthy tissues, as they induce a pro-inflammatory response and have been shown to inhibit axonal regeneration [30]. Conversely, Th2-activated microglia/macrophages (M2) have been considered protective, as they have a role in suppressing the pro-inflammatory response by producing anti-inflammatory mediators [30, 31]. Following spinal cord injury, there is evidence suggesting that the M1 response prevails over a more transient M2 response, and this observation has been proposed to contribute to the poor regenerative capacity of the spinal cord following injury [30, 31]. Consistent among various in vitro and in vivo studies, including spinal cord and peripheral nerve injury models, are reports of reduced levels of pro-inflammatory cell mediators, including as IL-6, iNOS, MCP-1, IL-1β and TNFα in response to treatment with various wavelengths including 633 nm [36], 660 nm, 780 nm [37], 810 nm [16] and 950 nm [14]. Coincidently, these pro-inflammatory cell mediators are secreted by M1 cells; thus, we were curious to examine the effect of light treatment on microglia/macrophage populations.

There are various wavelengths used throughout the literature which demonstrate biological effects. In an attempt to find the better wavelength option for treating nervous system injuries, one study compared the effects of two wavelengths in a variety of CNS injury models, to find that 670 nm treatment resulted in better outcomes for a number of parameters when compared to 830 nm [9]. Our aim therefore was to evaluate the effect of the 670 nm wavelength following spinal cord injury on a variety of functional parameters, namely the development of hypersensitivity to innocuous stimuli (allodynia), as well as on (tactile) sensory pathway conduction and locomotor recovery, and to see if there were alterations to the M1/M2 sub-populations. We found that red light treatment significantly reduced the severity of hypersensitivity while improving sensorimotor function and that these improvements were preceded by an anti-inflammatory microglia/macrophage cell population in the injury zone.

## Methods

### Hemicontusion spinal cord injury

All animal work was approved by the ANU Animal Experimentation Ethics Committee. Hemicontusion spinal cord injuries were performed on 7-week-old Wistar rats under isoflurane (1.7–2.3 % *v*/*v*) anaesthesia. Following hair removal, a laminectomy of T10 vertebral body and removal of dura and arachnoid was performed, followed by a spinal cord hemicontusion using a customized impactor system [38] comprising of a cylindrical 10 g weight with a 1-mm diameter tip that was guided onto the right dorsal horn and dropped from 25 to 50 mm above the spinal cord.

### Treatment and experimental groups

**Fig. 1:**
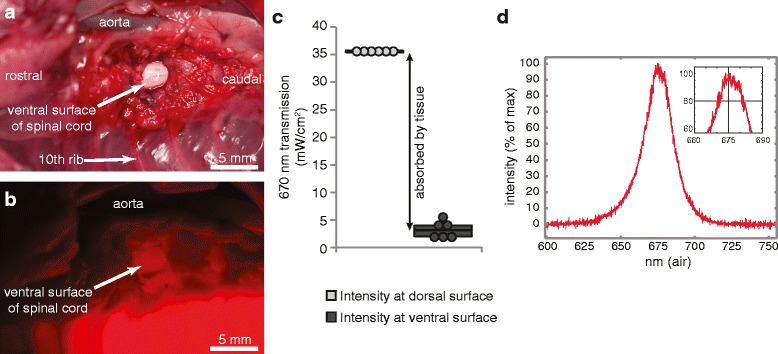
Externally applied red light penetrates through the entire rat spinal cord. **a** Photograph shows the ventral surface of the spinal cord following removal of the T10 vertebral body in a cadaver rat. Topography of the vertebral column is shown centred around the 10th vertebral body under normal light conditions. **b** The identical region as shown in **a**, with a 670 nm LED array light source (35 mW/cm^2^) placed directly on the dorsum of the animal and with ambient lights switched off. Note the visible red light illuminating from the ventral surface of the cord (exposed, *arrow*) indicating excess penetration through dorsal layers of the hair, skin, muscle, bone and spinal cord. **c** Intensities measured by a 670 nm power meter are shown for six freshly sacrificed cadaver rats (each *dot* represents the mean of triplicate readings). Readings shown are taken at the light source (through the Perspex restraining box, intensity at dorsal surface) and at the ventral surface of the spinal cord as shown by the *white arrow* in **b** (intensity at ventral surface). *Black arrow* indicates proportion of light absorbed and/or scattered by intervening tissues. **d** Spectral analysis of the light source indicating central frequency of 675 nm

Injured animals were divided into 670-nm-treated (SCI+670) and sham-treated (SCI) groups. SCI+670 rats received 30 min of 670 nm irradiation commencing 2 h after surgery and then every 24 h after locomotor assessment for the remainder of the recovery period. A commercially available 670 nm LED array (WARP 75A, Quantum Devices, Barneveld, WI; 75 mm^2^ treatment area) was used for treatment. Spectral characteristics and power output (Fig. 1) of the LED were measured using a spectrometer (CCS175, Thorlabs) and custom made power meter that was calibrated against a commercially available power meter (PM100D, ThorLabs). Treatment was delivered through a transparent treatment box which was used to confine the animal within its home cage. This resulted in a 7-mm distance between the dorsal surface of the animal and the LED array and delivery of 35 mW/cm^2^ (fluence = 63 J/cm^2^) of 670 nm at the contact surface of the animal’s dorsum. SCI rats (*n* = 29) were restrained in the identical way as the SCI+670 group (*n* = 29), but without the LED device switched on to control for 30 min restraint in the transparent treatment box. Three additional control groups were included: an intact uninjured group (control; *n* = 7) was untreated and did not receive any sham operations or sham treatment; a sham-injured group (shamSCI; *n* = 8) underwent the spinal surgery, but without the contusion, and was subjected to sham treatment; a sham-injured 670-nm-treated group (shamSCI+670; *n* = 10) underwent spinal surgery without the contusion and received daily 30 min treatments.

### Light penetration

Uninjured, unshaven animals (*n* = 6) were euthanised with sodium pentobarbital solution (325 mg/ml; Virbac; dosage, 100 mg/kg). The overlaying heart, great vessels and muscles were detached from the anchoring connective tissues and retracted to the side to expose the underlying vertebral column. The T10 vertebral body was eroded with a dental drill to expose the spinal cord from the ventral surface. The cadaver was placed on its back in an inverted transparent treatment box so that the dorsum of the cadaver could be positioned over the 670 nm LED array and the ventral surface of the rat was accessible to enable placement of a custom-built light measuring device. This device comprised of a photodiode chip (surface area, 0.62 mm^2^; maximal response (>95 %) to 630–685 nm; ODD-660W, Opto Diode Corp.) that was fixed to the bottom of an aluminium cylinder (height, 7.0 mm; external diameter, 8.7 mm). The top of the cylinder was sealed with a glass coverslip, and the entire probe was painted with black paint but leaving a small circular window (4.0 mm diameter) centred over the chip sensor. This left a ~2.4-mm lip between the external edge of the glass window and the external circumference of the cylinder. When pressed onto the ventral surface of the spinal cord, no light could penetrate from the side because the chip was located 7.0 mm behind the 4.0-mm aperture; thus, only light rays between 71° and 90° are able to reach the surface of the sensor; angles deviating from 90° do not hit the entire surface of the photosensitive diode and therefore contribute less to the total power reading. The signal from the probe was amplified by a custom amplifier built for purpose. The key component was the logarithmic converter amplifier (AD8304, Analog Devices). The readings were then calibrated against a commercially available light power meter tuned at 670 nm (PM100D ThorLabs) by producing a calibration table for different radiant power (controlled by distance from the light source) and subsequently converted into intensity (power/unit area). The probe was used to determine light intensity from the 670 nm array through (i) the treatment box, (ii) the spinal cord and dorsal overlying structures and (iii) the equivalent space through the air to provide a measure of attenuation over the distance of the light path. Prior to activating the LED, ambient lights were switched off; however, we also confirmed that no photons were detected by the light meter with the ambient lights on. Three repeat readings were acquired for each measurement.

Example images were obtained with a D1X Nikon (5.3 megapixels) camera and 120-mm lens (Medical NIKKOR) with a ×2 adaptor and built in ring flash. Images were captured with both the ambient lights and LED array on and then repeated in the same position with the ambient lights off.

### Temperature measurement

A temperature probe (ML309/MLT422, ADInstruments) connected to a data acquisition system (PowerLab 26T, LabChart v7.3.7, ADInstruments) was attached to the dorsum of the animals prior to, and 2 min after sham or light treatment on consecutive days from four sham- and four light-treated rats.

### Sensitivity assessment

Sensitivity assessment was carried out on day 7 post-injury prior to locomotor and electrophysiological assessments. To assess hypersensitivity, a nylon filament (OD: 1.22 mm) was used to deliver innocuous tactile stimuli over six defined regions over the animals’ dorsum: Above-Level (dermatomes C6-T3), At-Level (dermatomes T9-T12) and Below-Level (dermatomes L2-L5) on ipsi- and contralateral sides relative to the injury. The boundary for each of the six regions was marked on the animals’ back, and 10 consecutive innocuous “pokes” were delivered in each boundary at an inter-poke-interval of approximately 1–2 s, or until the animal recovered from movement evoked from the previous poke if longer than 2 s. Prior to testing, the operator practiced the stimulus procedure. This ensured that each poke was as brief as possible, that the filament landed normal (90°) to the skin surface and that the final position of the filament handle was approximately half the distance to that of the distance at initial contact of the filament. This protocol ensured pokes of consistent duration and maximum force which was confirmed using a weighing balance (maximum bending force: 2.86 ± 0.09 g; *n* = 10 pokes). During sensitivity testing, animals were “semi-restrained” in a V-shaped plastic box. This restricted the animal’s ability to avoid the testing procedure and thereby facilitated the operator’s accuracy of each poke but enabled sufficient movement for the animal to display behavioural responses of interest. Testing was recorded using a webcam (Logitec HD Pro C920). Videos were assessed blind to the observer in slow motion play back by evaluating the response to each innocuous poke that was graded into one of four categories as (I) no response; (II) mild response characterised by acknowledgment of the stimulus, head turns, brief shuddering of the contacted skin, but no obvious pain avoidance behaviours; (III) medium response, characterised by moderate signs of pain perception, including moderate avoidance attempts by moving away from the stimulus and (IV) severe response, characterised by severe signs of pain perception, including attacking the stimulus and “desperate” avoidance attempts and escape behaviours including jumping, running, writhing or audible vocalization. The four categories, I–IV, were chosen because these behaviours are easily distinguishable. The frequency of each response category was multiplied by a weight; categories I–IV were multiplied by 0, 1, √2 and 2, respectively, to provide greater separation between ordinal pain behaviours between non-painful and painful [39], as well as to help minimise heteroscedasticity of the data. The sum of the 10 weighted responses provided a regional sensitivity score (RSS) for each region. This paradigm enables high-resolution measures of sensitivity to 10 innocuous pokes with each possible RSS ranging between 0 and 20. Scores from ipsi- and contralateral regions were pooled to determine level sensitivity scores (LSS) above, at and below the level of injury. An cumulative sensitivity score (CSS) was derived for each animal by summing the RSS from all six regions; the maximum CSS possible is therefore 120. The hypersensitivity threshold was defined by the mean + 2 standard deviations (confidence interval of 95.5 %) of CSSs calculated from uninjured intact rats (control group).

### Somatosensory assessment

Animals were anaesthetised with urethane (12.5 % *w*/*v*; 1.4 g/kg; i.p.) and maintained at 37 °C on a heating mat. A tracheotomy was performed, and animals were placed in a stereotaxic frame. The gracile nuclei were exposed through the foramen magnum by head flection and removal of overlying muscles and meninges. Both left and right sciatic and sural nerves were exposed by the removal of the overlying skin followed by a splitting incision of the gluteus maximum and semimembranosus muscles, respectively. The exposed nerves were isolated from adjacent connective tissues and bathed in paraffin oil. Silver wire bipolar hook electrodes were used to stimulate sural nerves, and a single hook silver wire electrode was used to record from sciatic nerves to ensure complete recruitment of all sural nerve fibres upon electrical stimulation (square wave pulse, 0.5–1.1 mA, 0.05 ms). A platinum wire electrode was used to record from a single midline position on the brainstem at a location that was established to provide evoked potentials of equal magnitude and latency from left and right sural nerve stimulation. Thirty-three individual evoked potentials were recorded and averaged from the sciatic nerve and the brainstem in response to repeated sural nerve stimulations. Signals recorded from the brainstem were then processed offline (MATLAB, MathWorks). The averaged signal was band-pass filtered (500–3350 Hz) and response magnitudes calculated from the integral of rectified signals (integral limits: 5.00 ms before and 8.75 ms after the primary peak) after subtraction from baseline levels obtained prior to the stimulus. Latency was measured from the filtered signal where it first exceeded 3 standard deviations (confidence interval 99.7 %) of background levels.

### Locomotor assessment

Prior to surgery, animals were trained to run along an 80-cm custom build transparent walking-track with mirrors that reflected left and right sides and underneath of the animal. This enabled exquisite locomotor detail from all sides of interest to be video captured simultaneously from a single viewpoint. 2 h following surgery, initial recordings of animals running three consecutive times down the walking-track were acquired with a digital camera (Sony, NEX-VG20EH) at 50 frames per second, which provided adequate data for detailed gait analysis. Recordings were repeated every 24 h post-surgery for 7 consecutive days. Each video file was coded and assessed blind by one assessor. The BBB locomotor scale [40] for the left and right hind-limbs was used to generate locomotor scores from video files assessed in slow motion.

### Immunohistochemistry and TUNEL

Animals from both groups (SCI, *n* = 15; SCI+670, *n* = 15) were divided into three recovery time points and sacrificed at 1, 3 and 7 days post-injury. At the end of designated recovery periods, animals were transcardially perfused with saline and 4 % buffered paraformaldehyde (*w*/*v*). Harvested spinal cords were cryoprotected in 30 % sucrose (*w*/*v*), cryosectioned at 20 μm in the longitudinal plane using a Leica CM1850 cryostat, and dorsal sections labelled with primary antibodies (1:200) against rat CD68 (ED-1 clone, MAB1435, Millipore), and Arginase-1 (AB60176, Abcam) or CD80 (AB53003, Abcam) to quantify microglia/macrophages (ED1^+^) and polarized subtypes M1 (CD80^+^ED1^+^) and M2 (Arginase1^+^ED1^+^), respectively. Tissue was subsequently incubated with the appropriate secondary antibodies (1:1000, Invitrogen, Alexa 594 conjugated chicken anti-goat #A21468, Alexa 488 conjugated goat anti-mouse #A31619, Alexa 594 conjugated goat anti-mouse #A31623, Alexa 488 conjugated donkey anti-rabbit #A21206). Slides were then incubated in Hoechst solution (2 μg/ml Sigma-Aldrich). Standard immunohistochemical controls were included.

To detect cells undergoing apoptosis/necrosis, a TUNEL assay was performed. Slides were incubated with 1:10 Terminal Deoxynucleotidyl Transferase (TdT) buffer (125 mM Tris-HCl, 1 M sodium cacodylate, 1.25 mg/ml BSA, pH 6.6) for 10 min and then 1-h incubation at 37 °C with reaction mixture [0.5 enzyme unit/μl TdT (Roche Applied Science) and 2.52 μM Biotin-16-dUTP (Roche Applied Science) diluted in 1:10 TdT buffer]. This was followed by 15 min incubation in 1:10 saline sodium citrate (SSC) buffer (175.3 mg/ml sodium chloride, 88.2 mg/ml sodium citrate, pH 7.0) and blocked with 10 % normal goat serum in 0.1 M PBS for 10 min before incubating with secondary antibody in 0.1 M PBS (1:1000 dilution, Invitrogen, Alexa 488 conjugated streptavidin S11223) at 37 °C for 30 min.

All image analysis was performed blind to the experimental group. 2D images were constructed from three colour channel (red, green and blue) images acquired from a LED fluorescent microscope (Carl Zeiss Colibri) with a ×20 objective and digital camera (AxioCam MRc 5) with all settings kept constant for each channel. Cells with co-labelling were quantified with ImageJ (v1.46r) using the Cell Counter plugin that enables the placement of different classes of markers onto an image. Cytoplasmic markers, a class for each channel, were used to tag positive label in a single focal plane for all green and red channels that were examined independently. To define ED1^+^ cells, the accompanying DAPI^+^ nucleus (blue channel) was tagged for cells where ED1 staining was clearly complementing the DAPI surface profile. Double-labelled cells (i.e., ED1^+^Arginase1^+^ or CD80^+^) were evaluated by scrutinising all tagged DAPI^+^ cells for co-labelling in red and green channels. These cells were tagged again with another marker class. All markers were automatically quantified for each class by the software. Cells out of focus were not included. Cell counts were obtained from dorsal horn regions with viable tissue and quantified as the mean of duplicate images, each covering a minimum area 0.05 mm^2^. The areas of interest were defined and quantified prior to cell quantification and included the dorsal horn grey matter region as well as the white matter in the surrounding dorsal columns and lateral funiculus. Cell quantification is expressed as the number of cells per unit area (mm^2^).

### Statistics

All data expressed as boxplots with individual data points in figures or as mean ± SEM in the main text, unless otherwise stated. Boxplots indicate the median (thicker line), upper and lower quartiles with whiskers extending to maximum and minimum values excluding outliers (more than 1.5 times respective quartiles). Statistical analysis was carried out using R or MATLAB, and a criterion alpha level of 0.05 was adopted as statistically significant. Data sets were tested for normality and homoscedasticity, and *t* tests and linear mixed models (multi-factor ANOVA) were applied for normally distributed data (indicated by *) or Wilcoxon rank-sum (indicated by †) where data was not normally distributed.

## Results

### Red light penetrates the spinal cord

We first set out to demonstrate that red light can pass through superficial and deep structures underlying the dorsal exterior surface and penetrate the entire spinal cord (Fig. 1). The penetrating light could be seen with the naked eye (example, Fig. 1a, b). The dorsal surface of uninjured rats (*n* = 6) was exposed to the LED array and 670 nm light intensity measured at the light source surface through the transparent treatment box which directly contacts the rat dorsum during treatment (Fig. 1c, intensity at dorsal surface; 35.4 ± 0.05 mW/cm^2^) and the ventral surface of the spinal cord, where light had to pass through an additional ~10 mm of the animals’ tissues from dorsal surface (Fig. 1c, intensity at ventral surface; 3.2 ± 0.6 mW/cm^2^). These data show that 91.1 ± 1.8 % of the light from the LED array was absorbed/dispersed by the tissues between the dorsal surface of the animal and the ventral surface of the spinal cord (Fig. 1c, black arrow). To indicate the approximate attenuation over the distance of light travelling from the light source through to the ventral spinal cord surface, we measured the intensity at the approximate distance (10 mm) through the air (33.0 ± 0.5 mW/cm^2^). This demonstrated that the expected attenuation (~7 %) of light is negligible over the distance required to travel to the ventral surface of the cord.

### Surface temperature changes following light treatment

We measured the surface temperature of rats directly before and 2 min after treatment. Twenty-seven readings from sham-treated and 25 readings from light-treated animals were acquired from four animals in each group over consecutive days of treatment. While there was no significant difference in the surface temperature of sham-treated animals (before, 33.6 ± 0.23 °C; after, 33.6 ± 0.25 °C), there was a small but significant increase 2 min after light treatment (before, 32.8 ± 0.36 °C; after, 33.5 ± 0.22 °C; *p* = 0.038, paired *t* test).

### Red light reduces allodynia following spinal cord injury

**Fig. 2:**
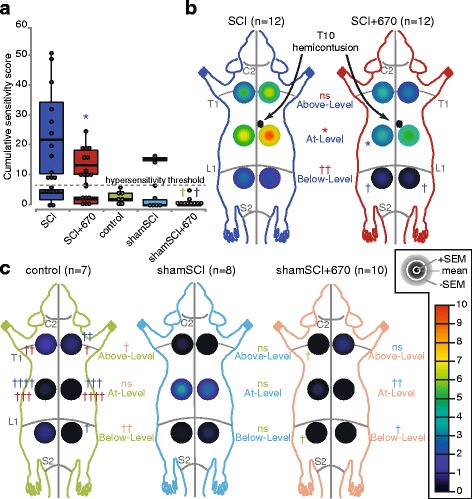
Hypersensitivity is reduced by red light treatment at 7 days post-T10 hemicontusion spinal cord injury. **a** CSSs (see the “Methods” section) for all groups are separated by the hypersensitivity threshold (6.9; indicated by *dotted green line*) into normosensitive (CSS < hypersensitivity threshold) and hypersensitive (CSSs > hypersensitivity threshold) subpopulations. **b** RSSs in hypersensitive sham-treated (SCI, *dark blue*) and 670-nm-treated (SCI+670, *dark red*) spinal cord injured animals (location of injury indicated). RSSs are represented as the mean ± SEM (colour-coded according to the insert: mean + SEM, mean, and mean − SEM concentrically represented) for the six tested regions (left and right sides; “Above-Level”, “At-Level” and “Below-Level” relative to the injury). RSSs are overlayed on schematic representations of the rat dorsum, with C2, T1, L1 and S2 dermatomes, and the midline, indicated (*grey*). Individual RSSs and LSSs are compared between hypersensitive subpopulation of the two groups. **c** RSSs shown for normal uninjured rats (control, *green*), sham-injury + sham-treatment (shamSCI, *light blue*, data includes both normo- and hypersensitive subpopulations), and sham-injury + 670 nm treatment (shamSCI+670, *light red*). Pairwise statistical comparisons are indicated for RSSs and LSSs by respective group colours. Note: statistical comparisons of CSSs from shamSCI+670 group in (**a**) is to the normosensitive subpopulation of SCI (indicated in *dark blue*) and to control groups (indicated in *green*); Statistical comparisons of RSSs from control group in (**c**) is to SCI (indicated in dark blue) or to SCI+670 (indicated in *dark red*) in **b**. *p < 0.05 (Student’s *t* test); ^†^ *p* < 0.05, ^††^ *p* < 0.01, ^†††^ *p* < 0.001, ^††††^ *p* < 0.0001, (Wilcoxon rank-sum); ns, *p* > 0.05; *n* values indicated

To examine the effect of red light on the development of neuropathic pain, we assessed sensitivity on six regions over the rat dorsum using a T10 hemicontusion spinal cord injury model that results in clear development of hypersensitivity in most animals within 7 days. The T10 spinal hemicontusion resulted in 63 % of animals (*n* = 12) developing hypersensitivity in both sham-treated (SCI, *n* = 19) and light-treated (SCI+670, *n* = 19) groups at 7 days post-injury. The hypersensitive subpopulation of rats from the SCI group had a mean CSS (SCI, CSS: 25.3 ± 4.5) that was 3.7 × the hypersensitive threshold (Fig. 2a). The mean CSS was significantly reduced by 40 % (SCI+670, CSS: 14.5 ± 1.6; 2.1 × the hypersensitivity threshold) in the hypersensitive subpopulation of rats from the SCI+670 group. Light treatment significantly reduced At- (T9-T12 dermatomes) and Below- (L2-L5 dermatomes) LSSs, which arose from contralateral At-Level and both ipsi-and contralateral Below-Level regions (Fig. 2b). Compared to the uninjured control group (control, Fig. 2c), sham injury without light treatment (shamSCI, *n* = 8) had no significant effect on LSS or RSS despite two sham-injured animals developing At-Level hypersensitivity. Light treatment of sham-injured animals (shamSCI+670, *n* = 10) resulted in significant reductions of At- and Below-LSS compared to the shamSCI group (Fig. 2c). Thus, while the incidence of hypersensitivity was not altered by red light, the level of hypersensitivity was markedly reduced At- and Below-levels in T10 contused light-treated allodynic animals. Red light also caused a significant reduction in sensitivity in 670-treated sham-injured animals (shamSCI+670, CSS: 0.8 ± 0.5) compared to uninjured control animals (control, CSS: 2.8 ± 0.8) as well as normosensitive spinal cord injured animals (SCI, CSS: 3.5 ± 0.9), even though these animals were not hypersensitive.

### Red light improves sensory conduction through dorsal column pathways

**Fig. 3:**
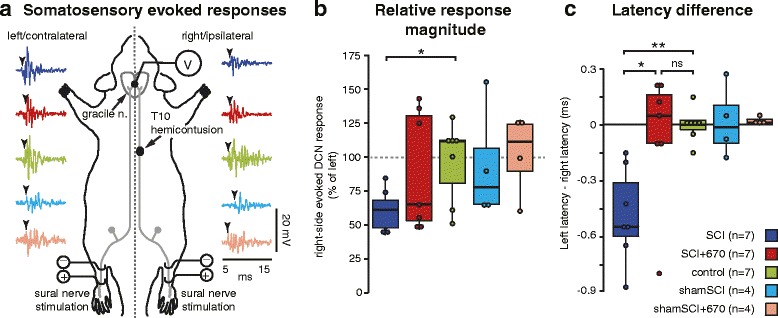
Dorsal column somatosensory functional deficits from T10 hemicontusion spinal cord injury is reversed by red light treatment. **a** Schematic of experimental paradigm for evaluating somatosensory (dorsal column pathway) functional integrity illustrating left and right dorsal column pathways (*grey*), T10 hemicontusion injury on right side, stimulation of sural nerves and location of recording electrode on midline of gracile nucleus. The same electrode position on the midline acquires somatosensory responses independently evoked from both left and right sural nerves, enabling direct comparable quantification of sensory pathways on both sides. Examples of responses (between 5 and 15 ms post-stimulus; 500–3350 Hz bandpass) evoked from left and right sides shown for respective groups (*colour-coded* as per legend in **c** and Fig. 2). *Arrowheads* indicate latency of response onset. **b** Quantification (integral of rectified signals) of gracile nucleus response magnitudes (right expressed as a percent of left). **c** Difference in latencies of evoked responses between left and right sides. Note magnitudes and latencies from intact animals are equal on both sides (control group). *p < 0.05; **p < 0.01, Student’s *t* test, Tukey’s post hoc in **c**

Could red light cause an anaesthetic-like effect on somatosensation that resulted in reduced sensitivity scores? To rule out the possibility that red light causes a reduced responsiveness to innocuous stimuli by bringing about a generalized inhibitory effect on somatic neural pathway conduction, we quantified the functional integrity of the sensory dorsal column pathway, at 7 days post-injury. The dorsal column pathways were activated by electrical stimulation of the left and right sural nerves, and a recording electrode was placed on the midline of the gracile nuclei (Fig. 3a). Stimulation of left and right nerves from control animals (*n* = 7) evoke responses of equal magnitude (Fig. 3b; right side: 101 ± 8 % of left side) and latency (Fig. 3c; left-right side latency difference: 0.09 ± 0.03 ms) on both sides when recorded from the same midline-positioned recording electrode, while sham-treated T10 hemicontusion spinal cord injury (*n* = 7) resulted in a 37 % reduction in magnitude (right side: 63 ± 16 % of left side) and a 0.48 ± 0.09 ms delay of the injured (right) pathway, when comparing the intact (left) side. Red light treatment (*n* = 7) rescued both the magnitude (Fig. 3b; right side: 93 ± 17 % of left side) and latency (Fig. 3c; left-right side latency difference: −0.05 ± 0.35 ms) deficits otherwise observed in the SCI group, indicating that red light treatment restored sensory pathway conduction, rather than impeding it. Furthermore, the rescued magnitude and latency deficits in the SCI+670 group indicates that their reduced sensitivity scores (Fig. 2) were unlikely to have resulted from a generalized reduction of somatic neural conduction.

We performed a variety of control experiments to validate our interpretations. There was no observable difference of conduction magnitudes or latencies in any of the sham-injured animals (shamSCI, *n* = 4; shamSCI+670, *n* = 4). There was no significant difference between gracile nuclei potentials evoked from the left sural nerve in any of the groups (SCI, 15.9 ± 1.8 μV · ms; SCI+670, 11.9 ± 2.4 μV · ms; control, 16.2 ± 3.6 μV · ms; shamSCI, 10.8 ± 2.6 μV · ms; shamSCI+670, 15.0 ± 2.8 μV · ms; *p* = 0.70, one-way ANOVA). Similarly, there was no significant difference of response latencies when evoked on the left side for all groups (SCI, 33.7 ± 0.3 μV · ms; SCI+670, 34.0 ± 0.4 μV · ms; control, 34.0 ± 0.4 μV · ms; shamSCI, 34.2 ± 0.5 μV · ms; shamSCI+670, 34.7 ± 0.3 μV · ms; *p* = 0.51, one-way ANOVA). These control experiments indicated that dorsal column pathway response magnitudes and latencies were similar between the different groups and largely unaffected contralateral to the injury.

### Red light improves locomotor recovery

**Fig. 4:**
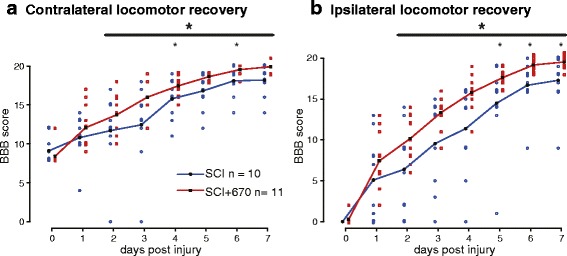
Locomotor recovery is improved by red light treatment following T10 hemicontusion spinal cord injury. Daily locomotor scores (BBB, see the “Methods” section) following a right-sided hemicontusion spinal cord injury are shown for the contralateral (**a**) and ipsilateral (**b**) sides. Red light treatment results in significant locomotor improvements on both sides over the period indicated by the *black bar* (*large asterisk*, linear mixed model with repeated measures). Point-wise comparisons between groups for individual time points are also shown (*small asterisks*, Student’s *t* test). Individual data points are presented as *open square* or *circular dots*; *lines* indicate the group means. *p < 0.05; **p < 0.01

Could red light treatment cause motor deficits and thereby result in reduced sensitivity scores? To rule out the possibility that the red light impeded the animals’ ability to perform escaping locomotor behaviours, daily locomotor recovery was examined blind to the experimental group (Fig. 4). We found that rather than impeding locomotion, the SCI+670 group (*n* = 11) demonstrated improved locomotor recovery as early as 2 days post-injury on the ipsilateral side and 3 days post-injury on the contralateral side compared to the sham-treated group (*n* = 10). Although a group effect of red light improvement was evident on the ipsilateral side (*p* = 0.026, linear mixed effects model with repeated measures), this failed to reach significance on the contralateral side (*p* = 0.055). There was a highly significant effect of time for both sides (*p* < 2e-16). Locomotor improvements observed in the SCI+670 group indicate that reduced sensitivity scores in light-treated animals (Fig. 2) could not have resulted from locomotor deficits.

### Red light reduces cell death at the injury zone

**Fig. 5:**
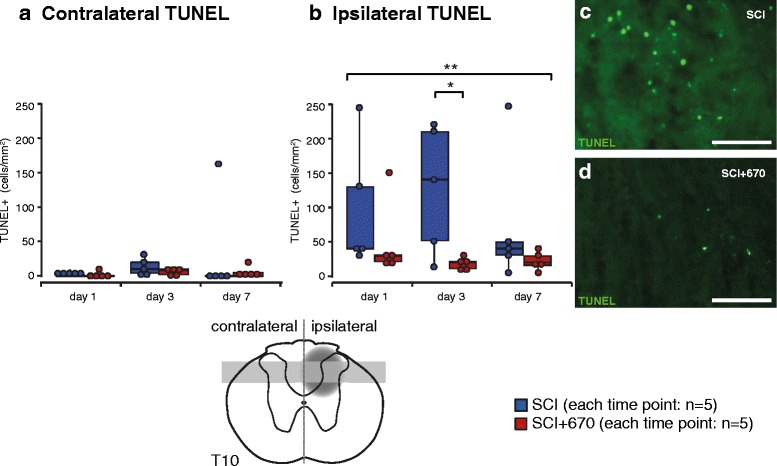
Cell death is reduced by red light following T10 hemicontusion spinal cord injury. Quantification of cells undergoing cell death (TUNEL^+^) contralateral (**a**) and ipsilateral (**b**) to the injury. Example images are from SCI (**c**) and SCI+670 (**d**) dorsal horn ipsilateral to the injury at 3 days post-injury. Schematic cross section of spinal cord (*bottom*) indicates location of injury (*dark grey* penumbra) and region of quantification (*light grey region*). *Scale bars*: 50 μm. *p < 0.05 (Student’s *t* test); **p < 0.01 (linear mixed model)

To examine the effect of red light on cell death following injury, the number of TUNEL^+^ cells was quantified at 1, 3 and 7 days post-injury in dorsal regions of the T10 spinal cord (Fig. 5, *n* = 5 for each time point). The SCI group resulted in an increased density of TUNEL^+^ cells in the dorsal spinal cord ipsilateral to the injury as early as day 1 (contralateral 1.5 ± 1.5 cells/mm^2^; ipsilateral 96.8 ± 41.1 cells/mm^2^), reaching maximum levels by day 3 (contralateral 13.1 ± 5.6 cells/mm^2^; ipsilateral 126.8 ± 41.5 cells/mm^2^). The contralateral side had much fewer cells where maximum levels were reached by day 7 (Fig. 5; contralateral 32.5 ± 32.5 cells/mm^2^; ipsilateral 74.2 ± 43.7 cells/mm^2^). Red light treatment resulted in a significant group reduction of TUNEL^+^ cells in the ipsilateral side, notably significant at the day 3 time point when TUNEL^+^ cells were maximal in the sham-treated group (1 dpi: 49.6 ± 25.2 cells/mm^2^; 3 dpi 18.2 ± 3.9 cells/mm^2^; 7 dpi 22.0 ± 6.1 cells/mm^2^). There was no significant difference in TUNEL labelling on the contralateral side between groups (1 dpi: 2 ± 2 cells/mm^2^; 3 dpi 6.2 ± 2.1 cells/mm^2^; 7 dpi 5.0 ± 3.9 cells/mm^2^).

### Red light reduces total activated microglia/macrophages but promotes the expression of the anti-inflammatory/wound-healing (M2) subtype

**Fig. 6:**
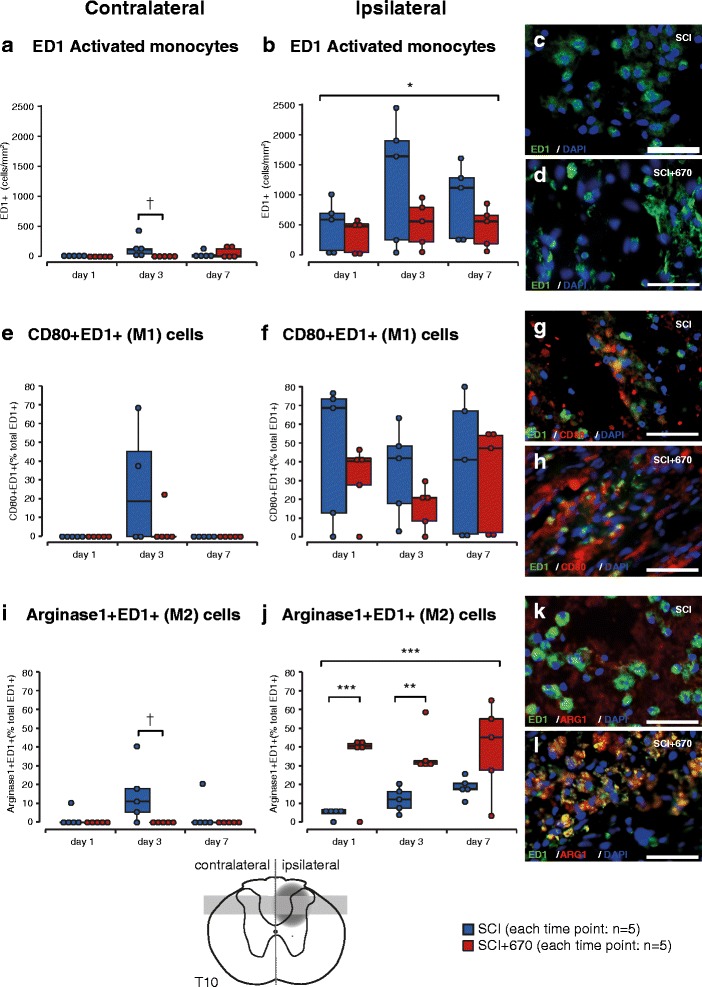
Anti-inflammatory microglia/macrophages are promoted early by red light treatment following T10 hemicontusion spinal cord injury. **a**–**d** Total activated microglia/macrophages (ED1^+^) per mm^2^ contralateral (**a**) and ipsilateral (**b**) to the injury and example images from SCI (**c**) and SCI+670 (**d**) groups. **e**–**h** M1 (pro-inflammatory) microglia/macrophages (CD80^+^ED1^+^ double labelled) expressed as a proportion of total ED1^+^ cells contralateral (**e**) and ipsilateral (**f**) to the injury and example images from SCI (**g**) and SCI+670 (**h**) groups. **i**–**l**: M2 (anti-inflammatory) microglia/macrophages (Arginase1^+^ED1^+^ double labelled) expressed as a proportion of total ED1^+^ cells contralateral (**i**) and ipsilateral (**j**) to the injury and example images from SCI (**k**) and SCI+670 (**l**) groups. All example images are taken from the injury zone of the dorsal horn at 7 days post-injury. Schematic cross section of spinal cord (*bottom*) indicates location of injury (*dark grey penumbra*) and region of quantification (*light grey region*). *Scale bars*: 50 μm. *p < 0.05 (linear mixed model); **p < 0.01, ***p < 0.001 (Student’s *t* test); ^†^ *p* < 0.05, ^††^ *p* < 0.01, ^†††^ *p* < 0.001 (Wilcoxon rank-sum)

Inflammation has long being implicated in the development of neuropathic pain [27]. We therefore quantified activated microglia/macrophages (ED1^+^ cells) at 1, 3 and 7 days post-injury in dorsal regions of T10 spinal cord (Fig. 6a–d, *n* = 5 for each time point). T10 spinal contusion resulted in an increase in ED1^+^ cell density as early as day 1 post-injury, reaching maximum levels by day 3 in the ipsilateral side. Maximum levels were also reached at day 3 on the contralateral side, but there were negligible ED1^+^ cells at days 1 and 7. Light treatment significantly reduced ED1 expression ipsilateral to the injury to approximately half that of the SCI group. Despite the low levels of ED1^+^ cells in the contralateral side, red light treatment also resulted in a significant reduction of ED1^+^ cells at the 3-day time point.

Microglia/macrophages can adopt pro- or anti-inflammatory states [30]. To determine the effect of red light treatment on the expression of pro-inflammatory (M1) cells, cells co-expressing CD80 and ED1 were quantified as a proportion of total ED1^+^ cells (Fig. 6e–h, *n* = 5 for each time point). The proportion of CD80^+^ED1^+^ cells ipsilateral to the injury was maximal at day 1 and remained greater than 40 % of the ED1 population at days 3 and 7 in more than half of animals. CD80^+^ED1^+^ cells were only found at day 3 on the contralateral side which coincided with the maximum number of ED1^+^ cells at that time point. Red light treatment did not have a significant impact on the proportion of M1 cells on either the ipsi- or contralateral sides. Note that no CD80^+^ED1^+^ cells were encountered at days 1 and 7 contralateral to the injury as ED1^+^ cells were also in small quantities at these time points (Fig. 6a).

To determine the effect of red light treatment on the expression of anti-inflammatory/wound-healing (M2) microglia/macrophages, ED1^+^ cells co-expressing Arginase-1 were quantified as a proportion of total ED1^+^ cells (Fig. 6i–l, *n* = 5 for each time point). In the SCI group, Arginase-1 expression increased with time ipsilateral to the injury (*p* = 0.0048, one-way ANOVA) but peaked at day 3 contralateral to the injury at the time when most ED1^+^ cells were present in that region. Ipsilateral to the injury, the SCI+670 group displayed significantly increased proportions of Arginase1^+^ED1^+^ cells from day 1, reaching approximately sevenfold that of the SCI group. This greater Arginase1^+^ED1^+^ proportion in light-treated animals was maintained at over one third of ED1^+^ cells for the entire duration investigated for the majority of animals. No Arginase1^+^ED1^+^ cells were detected contralateral to the injury in the SCI+670 group; however, there were very few ED1^+^ cells in this region (Fig. 6a). The group effect failed to reach significance contralateral to the injury (*p* = 0.0628) despite a significantly greater level of Arginase1^+^ED1^+^ cells in the SCI group.

## Discussion

We demonstrate that following spinal cord injury, 35 mW/cm^2^ of red (670 nm) light transcutaneously applied for 30 min/day for 7 days to the dorsal surface of rats is sufficient to reach the entire spinal cord and reduce the expression of pain behaviours. These reduced signs of allodynia are not due to sensorimotor deficits, as red light treatment improves both sensory and motor function. Alleviated hypersensitivity, improved tactile/proprioception (dorsal column) pathway functional integrity and locomotor functional outcomes are preceded by reduced numbers of dying cells and reduced numbers of activated microglia/macrophages around the injury zone. Furthermore, the proportion of anti-inflammatory/wound-healing (M2) microglia/macrophages is greatly enhanced by 24 h following light treatment.

We are confident that the power output of the red light was sufficient to penetrate the entire rat spinal cord as red light could be seen with the naked eye illuminating through to the ventral surface of the cord in the cadaver models. While penetration through to the rat spinal cord was achievable with an intensity of 35 mW/cm^2^, future studies would be required to determine the exposure parameters to achieve an equivalent level of irradiation in humans. Our finding of 91 % absorption (9 % excess penetration) is a conservative measure for two main reasons: (i) penetration measurements were obtained through the hair of unshaven animal cadavers (the injury site of all injured animals was shaven) and (ii) deoxygenated haemoglobin absorbs 670 nm significantly more than oxygenated haemoglobin [41, 42]. Measurements from freshly scarified animals are therefore likely to have increased levels of deoxygenated blood, and thus reduced penetration, compared to live animals. Another factor to consider is the small attenuation of light as a function of distance from its source. Our estimation indicates that over 93 % of the light would have reached the spinal cord ventral surface if no intervening tissues were present to absorb the light; thus, the effect of distance appears to be negligible. Nine percent excess penetration (i.e. 91 % absorption) from the surface of the skin (with hair intact) through all intervening tissue layers to the ventral surface of the spinal cord with a device delivering approximately 35 mW/cm^2^ is consistent with a recent study that demonstrated an excess penetration of 6.6 % through the surface of the skin and the muscle overlying the spinal cord with a device producing approximately 16 mW/cm^2^ in Fisher rat cadavers [9].

Temperature of sham-treated animals was not significantly different before and after treatment, while light-treated animals’ experienced a significant 1.2 °C increase. This increase does not exceed the normal range for rat tail skin temperature variations which have been reported to oscillate by ± 2 °C within a 2-h time frame [43]. However, as there was a small but significant temperature increase 2 min after light treatment, it is likely that there was a larger temperature increase during the 30 min treatment period. We therefore cannot rule out the possibility that temperature increases did not impact on our findings. Nevertheless, red light treatment does result in significant functional and cellular improvements, regardless if temperature is a contributing factor. If temperature increases were to contribute toward improved outcomes, it would be in contrast to studies of hypothermic treatment which propose superior outcomes following spinal cord injury [44–46]. As the mechanisms of action for light-treatment improvements remain to be elucidated, future investigations isolating the effect of temperature and light are warranted.

To our knowledge, our study is the first to report a red light-induced locomotor improvement following a spinal cord injury, which contradicts the only other study by Giacci et al. [9] that examined 670 nm on locomotor recovery with a daily dose of 28.4 J/cm^2^, an intensity of 15.8 mW/cm^2^ for 30 min, i.e. less than half the intensity of the present study. The compounded effect of reduced intensity and a more severe contusion injury in their study may explain this difference, and furthermore, suggests that matching the appropriate light dosage to the injury severity is of paramount importance.

Our T10 hemicontusion injury model resulted in allodynia within 7 days in a subset of animals. We are confident that our injury model results in neuropathic pain because hypersensitivity developed above and below the level, as well as contralateral to the injury, i.e. at dermatomes that receive their innervation from outside the injury epicentre. This observation is also consistent with findings from an investigation using a C5 hemicontusion injury model and which also found a subset of animals developing allodynia from 7 days post-injury that lasted for least 42 days [47]. Our observation of allodynia on the animals’ dorsum is also consistent with a T13 hemisection injury model that also results in clear development of hypersensitivity in most animals within 7 days and that remains persists for several weeks [48].

We found that red light treatment reduced the severity, but not the incidence of hypersensitivity at 7 days post-injury. As allodynia reached sensitivity levels of almost four times that of the hypersensitivity threshold, we would expect that a milder injury causing sensitivity scores closer to the hypersensitivity threshold boarder would result in a reduction of both the severity and incidence of hypersensitivity. The finding that the shamSCI+670 group demonstrated sensitivity significantly lower than that of the intact control and shamSCI groups was curious. Sham injury may have activated anti-nociceptive descending pathways such as periaqueductal grey/raphe magnus-mediated inhibition of dorsal horn nociceptive inputs [49]. Thus, endogenous central anti-nociceptive mechanisms, compounded by a red light-induced anti-inflammatory microenvironment, could be responsible for the sham-injured-light-treated animals expressing less sensitivity than observed in uninjured animals. This speculation warrants further investigation as red light-augmented relief from pain would have significant clinical relevance for post-surgical pain treatment.

Quantification of pain behaviours relies on sensory and motor functional integrity. We are confident that the reduced expression of allodynia in red light-treated animals was not due to diminished general somatic sensation or impeded motor function because red light improved, rather than impeded these parameters. Locomotor recovery and sensitivity testing was scored blind to the experimental group, and therefore, any subjective bias was eliminated. Sural nerve evoked somatosensory potentials in the gracile nuclei provided an objective and precise measure of somatic sensory functional integrity of both left and right dorsal column pathways. Sural nerves were stimulated to recruit all nerve fibres, and therefore, input to the spinal cord was identical on both sides, while the recording conditions on the midline of the gracile nuclei were also identical during the acquisition of evoked potentials elicited from pathways of both sides. Therefore, the only difference in the responses between the left and right sides was due to alterations within their respective dorsal column pathways. We further confirmed this by demonstrating equal magnitudes and latencies of somatosensory potentials in the gracile nuclei when evoked from left and right sural nerves of intact and sham-injured animals. Thus, our data indicates that reduced expression of behavioural signs of pain following red light treatment is unlikely to have resulted from locomotor or sensory deficiencies, but rather, represents a true reduction of pain experienced by the light-treated rats.

While our study is the first to demonstrate red light-induced pain relief from spinal cord injury, it is consistent with peripheral nerve injury studies that report pain relief accompanied by light-induced alterations to the inflammatory response [13–15, 50, 51]. The functional improvements found in red light-treated animals were observed after a significant reduction in cell death was apparent at day 3 post-injury, a time coincident with maximal levels of activated microglia/macrophages in the injury zone of sham-treated animals. Our observations of reduced ED1^+^ cells in 670-nm-treated animals is consistent with that found in retinal damage [7], as well as another study that demonstrated similar proportions of ED1 cell suppression lasting up to 14 days post-corticospinal tract lesion in rats that received daily 810 nm diode laser treatments [16]. In the latter study by Byrnes et al., they also demonstrated functional improvements of some motor tasks, also consistent with improved locomotor function observed in our study. While we cannot speculate on the mechanisms for 810 vs. 670 nm wavelengths to suppress microglia/macrophage activation and improve motor function, it is noteworthy that both wavelengths evoke peak levels of cytochrome C oxidase activity and ATP production [52]. However, wavelength (i.e. 660 vs. 780 nm) has been shown to alter the expression of inflammatory mediators expressed by activated pro-inflammatory microglia/macrophages [37], and light dosage has been shown to alter the balance of M1/M2 cell expression [53]. These in vitro studies suggest that other mechanisms, unrelated to cytochrome C oxidase, may influence the inflammatory microenvironment following light treatment. Furthermore, they highlight the necessity for thorough investigations to establish the therapeutic limits of any wavelength under investigation.

While others have demonstrated the impact of wavelength and dose on inflammatory cells in vitro [37, 53], to our knowledge, we are the first to demonstrate the effect of 670 nm light on the polarization of activated microglia/macrophages following spinal cord injury in vivo. The pattern and sequence of pro-inflammatory M1 (CD80^+^ED1^+^) cell activation, cell death, followed by anti-inflammatory/wound-healing M2 (Arginase1^+^ED1^+^) recruitment observed in sham-treated animals in our study is consistent with what is expected under conditions of spinal cord injury and repair [30, 31]. However, our data indicates that red light-induced reduction of cell death is preceded by the upregulation of M2 cells as early as 1 day post-injury. This is intriguing because the M2 cell expression preceded that of the M1 cells, indicating that red light drastically altered the normal sequence of inflammatory events. We therefore speculate that the early presence of protective M2 cells may have caused the reduced subsequent population of dying (TUNEL^+^) cells. Insufficient expression of the M2 subtype in spinal cord injury, in contrast to peripheral nerve injury, has been suggested to be a contributing factor to the poorer regenerative capacity and functional outcomes in spinal cord injury compared to peripheral nerve injury [30, 31]. The present study found that red light had a strong impact on promoting the M2 cell types as early as 24 h after treatment which was followed by reduced levels of cell death and subsequent improvement of sensory and motor functional outcomes thereafter. This is consistent with previous suggestions that enhancing the M2 population during recovery from spinal cord injury may indeed significantly contribute to improving functional outcomes following spinal cord injury [31].

## Conclusions

Modulating the severity of neuropathic pain by simply applying red light is an exciting prospect with great significant clinical relevance, despite not yet fully understanding the mechanism behind photobiomodulation. Our data demonstrates that red light treatment, a non-invasive and cost effective treatment, is able to significantly reduce the severity of pain in rats acutely after spinal cord injury, and these behavioural changes are accompanied by alterations to the alternatively activated macrophage population. Early pain intervention is considered important to avoid the prospects of developing chronic pain [54]. As 670 nm light therapy is FDA approved, it could be quickly adopted as an adjunct to early treatment of spinal cord injury. Not only could this minimise the severity of pain to sufferers, it may also provide collateral benefits which include functional improvements to other sensory/motor systems. However, translation to human patients requires further studies to determine exposure parameters such as the light intensity necessary to penetrate the human spinal cord.

## Abbreviations

ANOVA: Analysis of variance
CSS: Cumulative sensitivity score
BBB: Basso, Beattie and Bresnahan
CNS: Central nervous system
FDA: Food and Drug Administration
LED: Light-emitting diode
LSS: Level sensitivity score
M1: Th1-activated microglia/macrophages
M2: Th2-activated microglia/macrophages
PBS: Phosphate-buffered saline
RSS: Regional sensitivity score
shamSCI: Sham-treated sham-injured group
shamSCI+670: 670-nm-treated sham-injured group
SCI: Sham-treated spinal cord injured group
SCI+670: 670-nm-treated spinal cord injured group
SSC: Saline sodium citrate
TdT: Terminal deoxynucleotidyl transferase
Th1: T helper cell type 1
Th2: T helper cell type 2
TUNEL: Terminal deoxynucleotidyl transferase (TdT) dUTP nick-end labelling
